# A Novel Feeder-Free Culture System for Human Pluripotent Stem Cell Culture and Induced Pluripotent Stem Cell Derivation

**DOI:** 10.1371/journal.pone.0076205

**Published:** 2013-10-02

**Authors:** Sanna Vuoristo, Sanna Toivonen, Jere Weltner, Milla Mikkola, Jarkko Ustinov, Ras Trokovic, Jaan Palgi, Riikka Lund, Timo Tuuri, Timo Otonkoski

**Affiliations:** 1 Research Programs Unit, Molecular Neurology and Biomedicum Stem Cell Centre, University of Helsinki, Helsinki, Finland; 2 The Finnish Microarray and Sequencing Centre, Turku Centre for Biotechnology, University of Turku and Åbo Akademi University, Turku, Finland; 3 Children’s Hospital, Helsinki University Central Hospital, Helsinki, Finland; Massachusetts General Hospital, United States of America

## Abstract

Correct interactions with extracellular matrix are essential to human pluripotent stem cells (hPSC) to maintain their pluripotent self-renewal capacity during *in vitro* culture. hPSCs secrete laminin 511/521, one of the most important functional basement membrane components, and they can be maintained on human laminin 511 and 521 in defined culture conditions. However, large-scale production of purified or recombinant laminin 511 and 521 is difficult and expensive. Here we have tested whether a commonly available human choriocarcinoma cell line, JAR, which produces high quantities of laminins, supports the growth of undifferentiated hPSCs. We were able to maintain several human pluripotent stem cell lines on decellularized matrix produced by JAR cells using a defined culture medium. The JAR matrix also supported targeted differentiation of the cells into neuronal and hepatic directions. Importantly, we were able to derive new human induced pluripotent stem cell (hiPSC) lines on JAR matrix and show that adhesion of the early hiPSC colonies to JAR matrix is more efficient than to matrigel. In summary, JAR matrix provides a cost-effective and easy-to-prepare alternative for human pluripotent stem cell culture and differentiation. In addition, this matrix is ideal for the efficient generation of new hiPSC lines.

## Introduction

Human pluripotent stem cells (hPSC, including both human embryonic stem cells, hESC and induced pluripotent stem cells, hiPSC) require either a feeder cell layer or an extracellular matrix (ECM) coating to support their self-renewal, suggesting that signals originating from the ECM have a significant role in hPSC regulation. Consequently, there has been a growing interest in the extracellular milieu (or niche) of hPSCs. hPSCs are predominantly cultured on either mouse embryonic fibroblasts (mEF) or Matrigel, an extracellular matrix preparation isolated from mouse sarcoma [1-4]. However, undefined ECM preparations based on various animal glycoproteins and growth factors are not ideal for hPSC cultures as they may have unexpected and poorly controllable biological effects on the cells and furthermore, they cannot be used in eventual clinical applications.

A specialized extracellular matrix structure, basement membrane, underlies epithelial and endothelial cells, creating boundaries between different tissue types in a body [5,6]. Basement membranes consist of diverse protein and carbohydrate macromolecules that are secreted in cell type specific manner. Importantly, it has been shown that basement membranes not only provide mechanical support for tissues but also maintain tissue homeostasis [7,8]. The most important group of biologically active signaling proteins in basement membranes is laminins (lm). Laminins are composed of one alpha (α), one beta (β) and one gamma (γ) chain that are twisted together to form either a cruciform or a T-shaped structure. Currently, at least 15 different combinations (αβγ) of laminins are known [9-11].

We have previously shown that laminins-511 (α5β1γ1) and -111 (α1β1γ1), the two laminin isoforms expressed in early mouse embryos, are also synthesized by the hPSC cultures [12]. Our study also demonstrated that hPSCs utilize specific cell surface receptors when they adhere to the laminin isoforms. Crucially, we showed that undifferentiated hPSCs could be maintained on purified human lm-511 in defined culture medium. Various human recombinant proteins, including lm-511, vitronectin, fibronectin and their combinations have been shown to support hPSC maintenance [13-15]. However, large-scale purification or production of biologically functional human laminins by recombinant technologies is laborious and expensive. Therefore, here we have developed a feeder-free, cost-effective and user-friendly hPSC culture system that is based on the matrix secreted by human choriocarcinoma cell line JAR, producing high quantities of lm-511 and -111. Hereafter the matrix is called JAR matrix. 

## Materials and Methods

### Ethics Statement

The generation of human ES lines and their use in these studies was approved by the Ethics Committee of the Helsinki University Central Hospital (statement nr. 143/E8/01, on December 18, 2003).

Donors provided their written informed consent for participation. The procedure, generation and use of human iPS cells were approved by the Coordinating Ethics Committee of the Helsinki and Uusimaa Hospital District (statement nr. 423/13/03/00/08) on April 9, 2009.

The National Animal Experiment Board (http://www.laaninhallitus.fi/lh/etela/hankkeet/ellapro/home.nsf) authorized the use of mice in the teratoma assays. The animals were anesthetized by a mixture of Ketamine and Xylatsine and Carprofen was used as painkiller during the operation and day after. The animals were housed under controlled humidity, temperature, and light regimen and care was consistent with institutional and National Institute of Health guidelines. Teratoma growth was followed by palpation, and after seven to eight weeks, the mice were sacrificed using CO_2_ and the teratomas were dissected out.

### RNA Isolation and Reverse Transcriptase Reaction

Total RNA was isolated using NucleoSpin RNA XS kit (Macherey Nagel, Düren, Germany) without on-column DNase treatment. The DNase treatment (RQ1 RNase-free DNase; Promega, Madison, WI) was performed separately to confirm complete elimination of DNA. After the DNase treatment the samples were purified using NucleoSpin RNA Clean-up kit (Macherey-Nagel). One µg of total RNA was reverse transcribed into cDNA by M-MLV reverse transcriptase (Promega) in 20 µL reverse transcriptase reaction containing Oligo(dT)15 primers (Promega), the mix of four dNTPs, and RNAse inhibitor (RiboLock; Fermentas).

### RT-PCR

Total RNA was transcribed as described above. RT-PCR was performed by Maxima Hot Start Taq DNA Polymerase (Fermentas) as follows: enzyme activation step at 95°C for 4 minutes, followed by 40 cycles of 95°C, 30s/57°C or 60°C 30s/72°C, 30s and then the final elongation at 72°C for 7 minutes. The PCR products were separated in 2% agarose gel. The product for human lm α1 is 89 base pairs (bp), for human lm α2 169 bp, for human lm α3 211 bp, for human lm α4 226 bp, for human lm α5 97 bp, for human lm β1 247 bp, for human lm β2 298 bp, for human lm β3 201 bp, for human lm γ1 100 bp, for human lm γ2 197 bp and for human lm γ3 189 bp. Primers are shown in Table 1. We routinely run all our non-quantitative PCRs by using a pair of primers for the housekeeping gene, GAPDH, which recognize the possible genomic DNA contamination. The product for human GAPDH is 200 bp (+194 bp in case of genomic DNA contamination).

**Table 1:** *Primers used* in qRT-PCR and PCR.

**Gene**	**Forward primer**	**Reverse primer**	**Method**
Laminin alpha 1	AAGTGTGAAGAATGTGAGGATGGG	CACTGAGGACCAAAGACATTTTCCT	PCR
Laminin alpha 2	AAATGTACAGAGTGCAGTCGAGGTCA	CAGTGGATGCCTTCCACATTCACCTT	PCR
Laminin alpha 3	CACTGTGAACGCTGCCAGGAGGGCTA	CAGCTACCTCCGAATTTCTGGGGATT	PCR
Laminin alpha 4	CACTGTGAAAAGTGTCTGGATGGT	CAGGTGCTTCCAATGAGGAAGGGG	PCR
Laminin alpha 5	CCCAGCCCCTCTGTACCTC	GTTCACCGCCAGCCTCCTC	PCR
Laminin beta 1	AACTGTGAGCAGTGCAAGCCGTTT	CAACCAAATGGATCTTCACTGCTT	PCR
Laminin beta 2	CACTGTGAGCTCTGTCGGCCCTTC	CAAGGAGTGCTCCCAGGCACTGTG-3’	PCR
Laminin beta 3	CGGTTGGGTCAGAGTTCCATGC	GATCTGCTCCACACGCTTCTCC	PCR
Laminin gamma 1	CACAGAGCGGTTGATTGAAA	TGGGTCCCCTGTAGATTCTG	PCR
Laminin gamma 2	CCGTGCCAATCTTGCTAAAAG	GGGTCTTGTCACTGGCATCTG	PCR
Laminin gamma 3	CAGGCTCACCAGCCAGACG	GCAAGCAGCTCTGACAAGGTC	PCR
Sendai	GGATCACTAGGTGATATCGAGC	ACCAGACAAGAGTTTAAGAGATATGTATC	PCR
Oct4	TTGGGCTCGAGAAGGATGTG	TCCTCTCGTTGTGCATAGTCG	qRT-PCR
Sox2	GCCCTGCAGTACAACTCCAT	TGCCCTGCTGCGAGTAGGA	qRT-PCR
Nanog	CTCAGCCTCCAGCAGATGC	TAGATTTCATTCTCTGGTTCTGG	qRT-PCR
Sox17	CCGAGTTGAGCAAGATGCTG	TGCATGTGCTGCACGCGCA	qRT-PCR
Brachyury	GCATGATCACCAGCCACTG	TTAAGAGCTGTGATCTCCTC	qRT-PCR
Sox1	CATGCACCGCTACGACATG	AGGGCGACGCGCTCATGTA	qRT-PCR
Αlpha-fetoprotein	CGCTGCAAACGATGAAGCAAG	AATCTGCAATGACAGCCTCAAG	qRT-PCR
Albumin	GGAAAAGTGGGCAGCAAATGT	GGTTCAGGACCACGGATAGA	qRT-PCR

### Radioactive Labeling and Immunoprecipitation

The cells were labeled and the samples processed as described earlier [12]. Briefly, JAR cells were labeled overnight in culture medium devoid of serum containing 100 µCi ^35^S-methionine to label all the proteins produced by the JAR cells. Then, the medium and matrix produced by JAR cells were collected and processed as described. Lm α1 and lm α5 were immunoprecipitated from the culture medium and matrix by specific monoclonal antibodies (Table 2). Then, the immunoprecipitated proteins were separated in 5% polyacrylamide gels. Finally, the gels were dried onto Whatman filter paper and detected by autoradiography.

**Table 2:** Antibodies.

**Antibody**	**Clone/catalogue**	**Reference**	**Method**
SSEA3	MC-631/MAB4303	Solter and Knowles 1979; Peter Andrews, Sheffield, UK	FACS, ICC
SSEA1	MC-480/MAB4301	Millipore	FACS
TRA1-60	TRA1-60/MAB4360	Millipore	FACS, ICC,
H-type 1	Ab3355	Abcam	FACS
Oct4	Cat. H134	Santa Cruz	ICC,
Nanog	D73G4/Cat. 4903	Cell Signaling Technology	ICC
Sox17	AF1924	R & D Systems	IHC
Tuj1	Tuj1/Cat. MAB1195	R & D Systems	ICC, IHC
Vimentin	Cat. H84	Santa Cruz	IHC
Pax6	Cat. PRB-278P	Covance	ICC
Albumin	188835/MAB 1455	R & D Systems	FACS, ICC
Alpha-fetoprotein	A0008	DAKO	ICC
Alexa Fluor anti-mouse IgG 488	A21202	Life Technologies	ICC
Alexa Fluor anti-mouse IgM 488	A21042	Life Technologies	FACS, ICC
Anti rat IgM phycoerythrin	G53-238/Cat. 553888	BD Pharmingen	FACS
Anti rabbit IgG 594	A21207	Life Technologies	ICC
Anti mouse IgM	A21046	Life Technologies	ICC
Lm α5	4C7	[32]	IP
Lm α1	161EB7	[33]	IP

Abbreviations: FACS: fluorescence-activated cells sorting/flow cytometry; ICC: immunocytochemistry; IHC: immunohistochemistry; IP: immunoprecipitation.

### JAR Cell Culture

Human choriocarcinoma cells (HTB-144™, American Type Culture Collection; ATCC) were cultured on tissue culture dishes in RPMI -1640+GlutaMAX culture medium containing 10% fetal bovine serum (FBS; Promocell, Heidelberg, Germany). The cells were passaged by trypsin every three to four days.

### Acellular Matrix Preparartion

Approximately 1x10^4^ JAR cells per cm^2^ were plated on plastic culture dishes pre-coated with 0.1% gelatin (Sigma-Aldrich). The cells were allowed to grow for 48 hours, after which they were washed once with 1X phosphate buffered saline (PBS) and lysed by 1mM NH_3_ for 30 minutes at room temperature (RT). The plates were then carefully washed with 1X PBS for three to four times. Next, the pH of the plates was balanced by incubating the plates in basal culture medium (DMEM/F12+GlutaMAX;Life Technologies) at the cell culture incubator for over night. The plates were either used immediately or stored at 4°C, in 1X PBS or dried, for up to 4 months.

### hPSC Lines

Three pre-established hPSC lines were used in this study. hESC line FES 29 [16,17] has been derived and thoroughly characterized in our laboratory. hESC line H9 [18] was purchased from WiCell institute. The hiPSC line HEL 11.4 [19] has been generated and thoroughly characterized in our laboratory. For generation of HEL11.4 hiPSC line, fibroblasts of a healthy adult donor were induced to pluripotency by Sendai reprogramming kit (OCT4, SOX2, KLF4, C-MYC; CytoTune; Life Technologies).

### hPSC Culture

The hPSCs were cultured on tissue culture dishes coated with growth factor reduced Matrigel (diluted 1:200 in DMEM/F12+GlutaMAX; BD Biosciences, NJ, USA) or on the JAR matrix, prepared as described above. Defined, serum-free cell culture medium, StemPro (Life Technologies) was used as culture medium in all standard cultures. The hPSCs were passaged by collagenase IV treatment. The cells were incubated in collagenase IV (1 mG/mL; Invitrogen) for 4 minutes at 37°C and washed once using basal culture medium. Then, the colonies were detached by cell lifters, triturated into small clumps and plated on fresh Matrigel or JAR matrix plates in StemPro culture medium. Prior to culture on JAR matrix, the hPSCs had been cultured on growth factor reduced Matrigel, as described above. The early-passage hiPSC were propagated by manual cutting.

### qRT-PCR

For real-time SYBR Green qPCR, total RNA was reverse transcribed as described above. Each multiplication reaction run was made in duplicates. The reactions contained SYBR Green JumpStartTaq ReadyMix (Sigma-Aldrich), 1µL cDNA template, (RT-reaction), forward and reverse primers (Oligomer, Helsinki, Finland) and nuclease-free water (Amresco, Solon, OH) at 20µL. The reactions for the qPCR were prepared using a Corbett CAS-1200 liquid handling system and the qPCR was performed using Corbett Rotor-Gene 6000 (Corbett Life Science, Sydney, Australia) as follows: enzyme activation step at 95°C for 4 minutes following 40 cycles of 95°C, 20 s/57°C, 20 s/72°C, 20 s, followed by a melting step. Data was analyzed according to the comparative ∆∆Ct method (Applied Biosystems, User Bulletin 2). As a reference sample, we used optimized, in-house prepared positive control mix, which contains cDNA from undifferentiated hPSCs, spontaneously differentiated hPSCs as well as from isolated human pancreatic islets. The primer sequences are listed in Table 1.

### Immunocytochemistry

The cells were fixed by 4% paraformaldehyde and washed several times with 1X PBS. The samples were permeabilized by 0.5% Triton X-100-PBS for 7 minutes at room temperature (RT) and washed once with 1X PBS. Then, the samples were treated with Ultra V Blocking solution (Thermo Scientific, Waltham, MA, USA) to prevent unspecific binding of antibodies. Primary antibodies (Table 2) were diluted into 0.1% Tween-PBS and incubated on the samples over night at 4°C. Next, the samples were thoroughly washed three times with 1X PBS. The secondary antibodies (Table 2) were diluted 1:500 in 0.1% Tween-PBS and incubated on the samples for 30 minutes, at RT, in dark. Finally, the samples were washed as above and mounted in 4',6-diamidino-2-phenylindole (DAPI) mounting medium (Vector Laboratories, Burlingame, CA, USA).

### CELL-IQ Imaging

The early hiPSC colonies were stained for presence TRA1-60 (Table 2) and taken into Cell-IQ cell imaging system (ChipMan Technologies, Tampere, Finland) to quantify the number of TRA1-60 positive colonies in each well.

### Flow Cytometry

The hPSCs were detached by TrypLE treatment for 2 minutes and washed in flow cytometry buffer (5% fetal bovine serum in 1X PBS). Then, the cells were incubated with the primary antibodies (Table 2) diluted in the flow cytometry buffer for 1 h on ice. Next, the cells were washed three times with flow cytometry buffer by centrifuging at 800 rpm for 5 minutes. The secondary antibodies (Table 2) were diluted in flow cytometry buffer and incubated on the cells for 30 minutes, on ice, in dark. The cells were washed as above and ran either fresh or fixed by paraformaldehyde (PFA) and run later by FACS Calibur (BD Biosciences) using CellQuestPro software (BD Biosciences).

The differentiated, hepatocyte-like cells were detached for 10 to 15 minutes in Trypsin-EDTA (Sigma-Aldrich, Aldrich). Next, the cells were washed and fixed by 4% PFA for 15 minutes at RT. Then, the cells were washed once in saponin-containing flow cytometry buffer (0,1% Saponin and 5% FBS (Promocell, Heidelberg, Germany) in PBS) prior to 60 min incubation with primary mouse anti-human albumin antibody (0,5 μg/1x10^6^ cells; R&D Systems). The cells were washed three times with saponin-containing flow cytometry buffer and incubated for 30 min with the secondary antibody. Then, the cells were washed and analyzed as above.

### Embryoid Bodies

The cells were detached by collagenase IV (1mG/mL), washed and triturated into small (approximately from 20- to 40-cell) clumps. The cells were grown on Ultralow attachment dishes (Corning, Tewksbury, MA, USA), in hPSC culture medium without basic fibroblast growth factor (20% KnockOut Serum Replacement, 1% non-essential amino acids, and 0.1mM 2-mercaptoethanol in KnockOut DMEM containing 1X GlutaMAX; all from Life Technologies) for 10 days. Then, the embryoid bodies were collected, washed once using 1X PBS and fixed with 4% PFA for 20 minutes at RT. The fixed samples were then washed several times by 1X PBS and embedded into 2% agarose gels, dehydrated and embedded into paraffin. The sections were then immunostained to detect endodermal (SOX17), ectodermal (BETA(III)TUBULIN) and mesodermal (VIMENTIN) derivatives. Shortly, the sections were deparaffinizated and the primary antibodies (Table 2) were incubated on the samples for over night at 4°C. Excess antibodies were removed by washing the samples three times by 1X PBS. Then, the samples were treated with the secondary antibodies for 30 minutes, at RT, in the dark. Finally, the samples were washed as above and mounted into DAPI mounting medium.

### Teratomas

The hPSCs were detached by collagenase IV treatment, washed and injected into nude mice testis. The tumors were harvested at 6-8 weeks after injection, fixed by 4% PFA and processed into paraffin sections. Histology of the tumors was examined after hematoxylin & eosin staining.

### Neuronal Differentiation

The cells were cultured on Matrigel and JAR matrix until 70-80% confluency. The cells were washed twice with 1X PBS and the neural differentiation medium (1X B27, 1X N2, 2µM dorsomorphin, 2µM SB431542 in DMEM/F12 containing 1X GlutaMAX) was changed on the cells (day 0). The pluripotent status of the cells was confirmed at day 0 by performing a flow cytometry analysis of the cells using antibodies recognizing TRA1-60 and SSEA3 epitopes. The neural differentiation medium was replaced daily for seven days, after which samples were collected for qPCR and immunocytochemistry analysis.

### Hepatocyte Differentiation

The cells were cultured on Matrigel and JAR-matrix in StemPro medium until 80-90% confluency. The cells were then washed twice in PBS and cultured for 24 h in RPMI-1640 + GlutaMAX medium supplemented with 2% B27 (both from Life Technologies), 100 ng/ml Activin A (a gift from Dr Marko Hyvönen), 3µM CHIR99021 (Stemgent, Cambridge, MA, USA) and 1 mM sodium butyrate (NaB;Sigma-Aldrich). After 24 h from the onset of differentiation NaB concentration was decreased to 0.5 mM and after 48 h the CHIR99021 was removed from the medium. Then the cells were cultured in this medium for the following three days with a daily media change. Differentiation from definitive endoderm (DE)-stage cells into hepatocyte progenitors was performed essentially as described by Hay et al in 2008 [20]. Briefly, the DE-cells were washed with PBS and cultured for five days in KO-DMEM media supplemented with 20% KO-SR, 1% NEAA, 0.1 mM 2-mercaptoethanol (all from Life Technologies), 1 mM L-Glutamine (Omega Scientific, Inc, Tarzana, CA, USA), and 1% DMSO (Sigma-Aldrich). For hepatocyte maturation, the cells were washed with PBS and cultured for eight to ten days in Leibovitz’s L-15 medium (Life Technologies) supplemented with 8.3% fetal bovine serum (FBS, Promocell), 8.3% Tryptose phosphate broth (Sigma-Aldrich), 10 µM Hydrocortisone 21-hemisuccinate (Sigma-Aldrich), 1 mM Insulin (Roche), 2mM Glutamine (Life Technologies), 25 ng/ml Hepatocyte growth factor, HGF (Peprotech, Stockholm, Sweden) and 20 ng/ml Oncostatin M (R&D Systems). Throughout the differentiation the culture medium was changed daily.

### Retroviral hiPSC Inductions

5x10^4^ human foreskin fibroblast (ATCC) cells were plated into each well of the tissue culture treated six-well plate. Inductions with retroviral transgene delivery were done using two bi-cistronic viruses encoding OCT4 and KLF4 in one virus and SOX2 and c-Myc in the other. Viruses were produced in 293-GPG packaging cell line and pooled media collected from 3 to 5 days after transfection was used to infect the fibroblasts twice. 3 days after the last infection cells were split and plated on either JAR matrix or Matrigel coated plates for comparison. For the pluripotency induction cell culture medium was changed to hESC medium ((KnockOut™ DMEM;Life Technologies) supplemented with 20% KnockOut™ serum replacement, 1% GlutaMAX, 0.1mM-mercaptoethanol, 1% nonessential amino acids (all from Life Technologies) and 6 ng/ml basic fibroblast growth factor (Sigma-Aldrich)) supplemented with 0.25mM sodium butyrate. Induction efficiencies were determined by counting TRA1-60 positive colonies from triplicate inductions at day 14. The hiPSC colonies were picked to passage 1 at day 14 post inductions.

### hiPSC Inductions with Sendai Viruses

Before the inductions, human foreskin fibroblasts (ATCC) were plated on cell culture dishes (approximately 1X10^4^ cells/cm^2^). Fibroblasts were induced to form pluripotent cells by a commercially available Sendai Reprogramming Kit (CytoTune; Life Technologies) that utilizes the four human transcription factors, OCT3/4, SOX2, KLF4 and C-MYC. The infections were performed according to the manufacture’s instructions. On day 7 post infection, the cells were passaged on JAR matrix and between days 16 and 19 post infections, the formed hiPSC colonies were picked on JAR matrix.

### Statistical Analysis

Student’s t-test with a 95% confidence level was used to calculate the significance of differences between cells cultured on Matrigel *vs.* JAR matrix in differentiation analysis, hiPSC induction efficacy and hiPSC colony adhesion at passage 1.

## Results

### JAR Choriocarcinoma Cells Secrete Laminins -511 and -111

**Figure 1:**
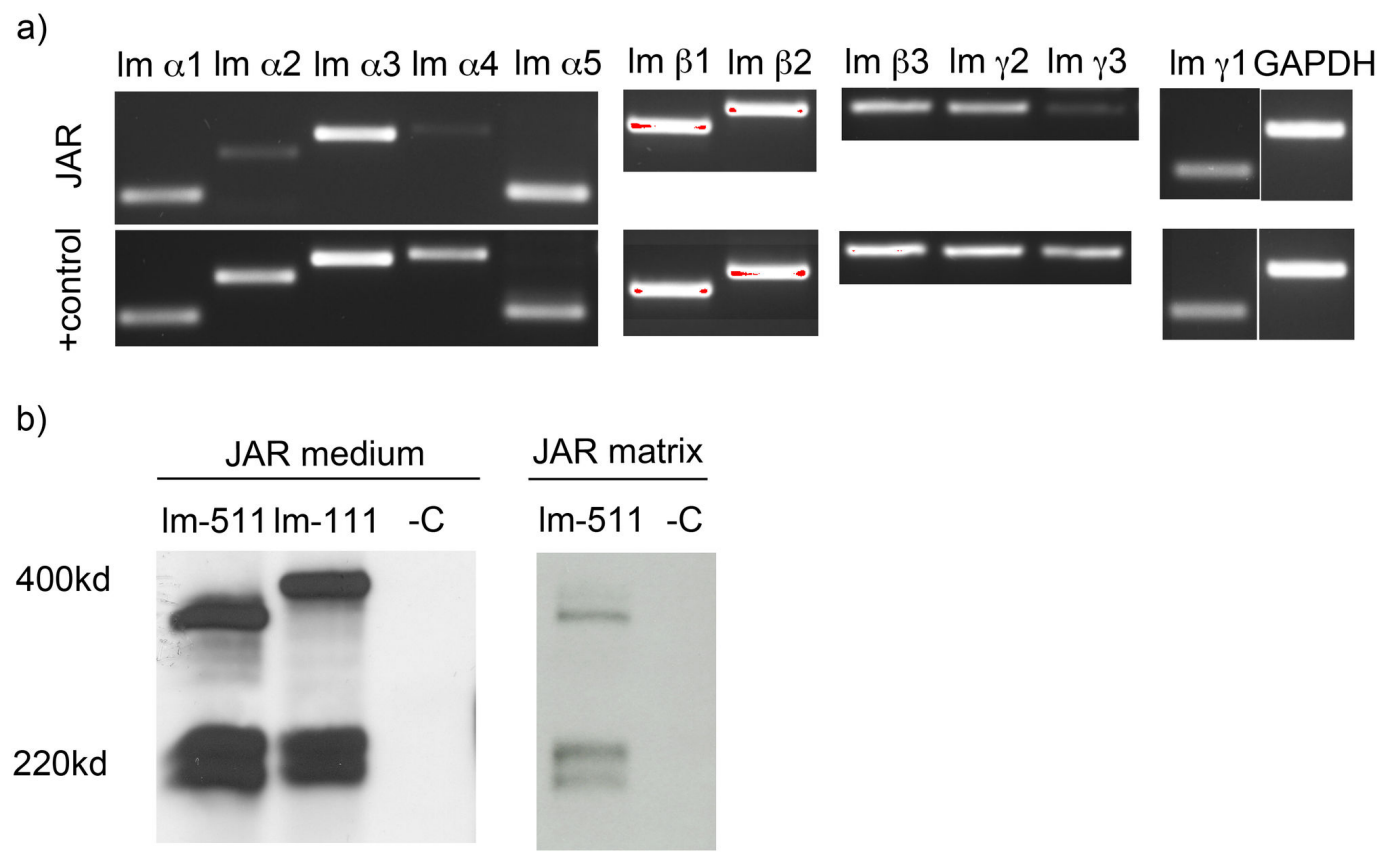
Expression and synthesis of laminins by JAR cells. A) JAR choriocarcinoma cells transcribe lm α1, lm α3, lm α5, lm β1, lm β2, lm β, lm γ2, and lm γ1 chains as shown by RT-PCR. As a reference sample, we used optimized, in-house prepared positive control mix, which contains cDNA from undifferentiated hPSCs, spontaneously differentiated hPSCs as well as from isolated human pancreatic islets. B) JAR choriocarcinoma cells were metabolically labeled with ^35^S and the cell culture supernatant and matrix deposited by JAR cells were immunoprecipitated using antibodies, which specifically recognize the human lm α1 (400 kDa) and lm α5 (350-380 kDa) chains. The cells showed abundant lm-511 and -111 synthesis.

To investigate the production of laminin subunits by JAR cells we performed RT-PCR and immunoprecipitation analyses. Figure 1a shows the mRNA expression of laminin alpha 1, alpha 3, alpha 5, beta 1, beta 2, beta 3, gamma 1, and gamma 2 chains. We next confirmed that the JAR cells synthesized lm-511 and -111 proteins by performing the metabolic, radioactive labeling of the cells, followed by immunoprecipitation of the lm α5 and α1 chains from the JAR cell culture medium by using monoclonal antibodies (Table 2). The results demonstrate that JAR cells abundantly secrete lm-511 and -111 isoforms into culture medium (Figure 1b; on the left). In addition, we confirmed that JAR cells deposit lm-511 into ECM (Figure 1b; on the right).

### The JAR Cell Culture Matrix Supports Long-Term Maintenance and Pluripotency of hPSCs

**Figure 2:**
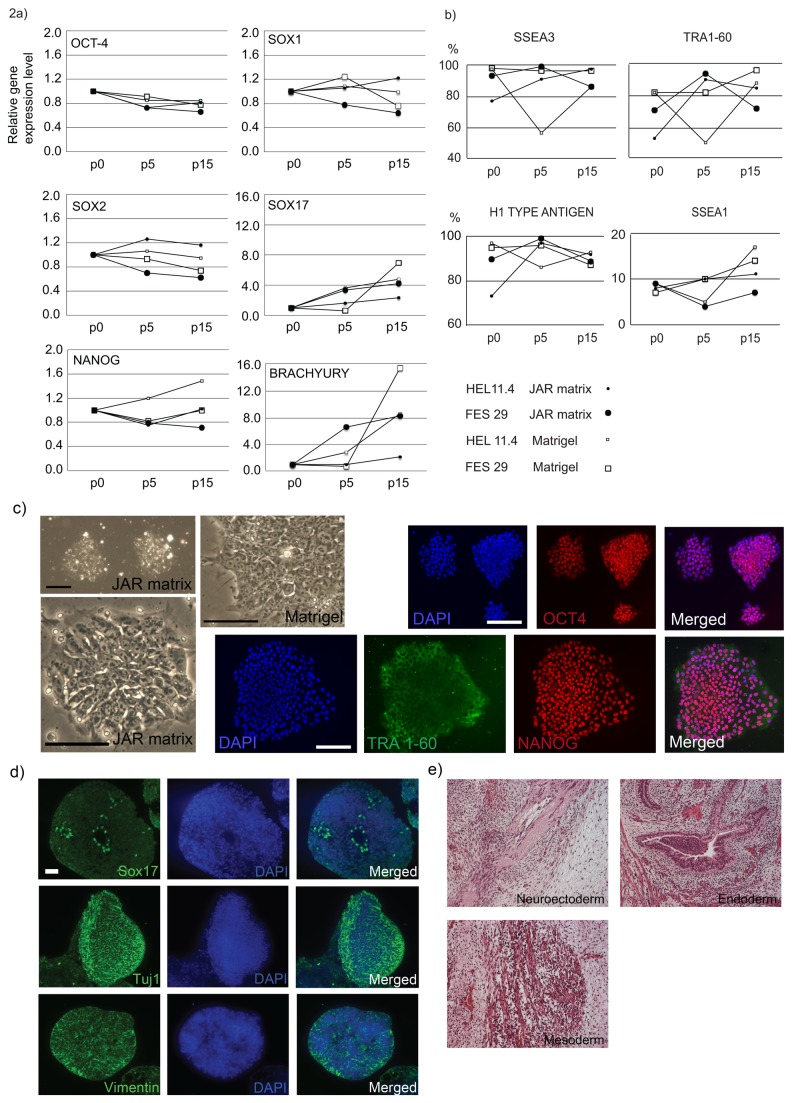
JAR matrix maintains hPSCs undifferentiated and pluripotent. A) Two hPSC lines, FES29 and HEL11.4, were cultured on JAR matrix or Matrigel for 15 passages and characterized in the beginning (p0), at p5 and at p15. Expression of genes typical for undifferentiated hPSCs (OCT4, SOX2, NANOG) and early differentiation markers (SOX1, SOX17 and BRACHYURY) were analyzed by qPCR. Each data point represents the pooled sample from five replicate plates per cell line. B) The hPSC lines were analyzed by flow cytometry in the beginning (p0), at p5 and at p15. The levels of SSEA3, TRA1-60 and H1 type antigen were high throughout the culture period, while the level of SSEA1 remained low, suggesting that pluripotent hPSCs were maintained on JAR matrix equally well as on Matrigel. Each time point represents the pooled sample from five replicate plates per cell line. C) Morphology of the hiPSC line HEL11.4 on JAR matrix and on Matrigel. Phase contrast micrographs and immunofluorescent staining of the hiPSC line HEL11.4 with antibodies recognizing OCT4, Tra1-60 and NANOG, after culture for 15 passages on JAR matrix. Scale bars: 500 µm for the upper phase contrast image of HEL11.4. on JAR matrix, others 100 µm. D) hPSCs formed typical embryoid bodies after 15-passage culture on JAR matrix. Presence of endodermal (SOX17), ectodermal (BETA(III)TUBULIN/TUJ1) and mesodermal (VIMENTIN) derivatives was detected by immunofluorescence in hiPSC line HEL 11.4. Scale bar 100 µm. E) Mature derivatives of all germ layers; neuroectoderm, endoderm and mesoderm in a teratoma obtained from the hiPSC line HEL11.4 after culture on JAR matrix for 15 passages. Objective magnification 40X.

We cultured one hESC line, FES29 [16] and one hiPSC line, HEL11.4 [19] in parallel for 15 passages and a second hESC line, H9 [18] for 12 passages on both JAR matrix and growth factor reduced Matrigel in a defined, serum-free culture medium, StemPro. At the end of culture, the cells were characterized and their differentiation capacity was evaluated with several methods. Figure 2 shows characterization of the FES29 and HEL11.4 cells cultured on either JAR matrix or Matrigel, for 15 passages. The cell lines maintained high expression levels of the pluripotency marker genes OCT4, SOX2 and NANOG on both matrices (Figure 2a). The expression level of some differentiation-related genes, such as SOX17 and BRACHYURY tended to slightly increase during culture but the changes were minimal on both matrices and even smaller on JAR matrix as compared to Matrigel (Figure 2a).

To quantify the proportion of the undifferentiated hPSCs we performed a series of cell surface marker analyses with flow cytometry on the cells cultured on JAR matrix or Matrigel. Figure 2b shows that the hPSCs maintained their undifferentiated state equally well on both matrices. At passage 15, both cell lines had more than 85% SSEA3 positive cells on JAR matrix and Matrigel. At the same time, 72% (FES29) and 85% (HEL11.4) of the cells were TRA1-60 positive on JAR matrix and 96% (FES29) and 85% (HEL11.4) of the cells were TRA1-60 positive on Matrigel (Figure 2b). We also analyzed the presence of the H type 1 antigen [19,21] on the hPSCs and found that it was present on approximately 90% of the cells, regardless of the cell line, passage or culture conditions. SSEA1 antigen, which refers to differentiation of the hPSCs, was present on 7% (FES29) and 11% (HEL11.4) of the cells that had been cultured on JAR matrix for 15 passages and on 14% (FES29) and 17% (HEL11.4) of the cells that had been cultured on Matrigel. figure S1 represents the flow cytometry histograms of the FES29 and HEL11.4 cells cultured on JAR matrix for 15 passages. Both of the cell lines maintained typical undifferentiated morphology and showed nuclear expression of the transcription factors POU5F1 (also known as OCT4) and NANOG and also showed presence of TRA1-60 antigen after being cultured on JAR matrix or Matrigel (Figure 2c shows the results for the hiPSC line HEL 11.4.).

The chromosomal integrity of the cells was studied by the novel method, KaryoLite [22]. The assay measures DNA copy numbers at the chromosome arm resolution utilizing bacterial artificial chromosome (BAC) probes immobilized onto color-encoded polystyrene microspheres distinguishable by fluorometry. The probes are targeted to proximal and terminal regions of each chromosome arm of metacentric and sub-metacentric chromosomes and q-arms of acrocentric chromosomes. As previous studies have indicated that the most common karyotype abnormalities in hESCs involve gains of whole chromosomes or telomeric regions of chromosomes, it is expected that the probes included in the KaryoLite™ BoBs™ cover the frequent and recurrent abnormalities [23]. The method is superior to the conventional karyotyping covering the genomic DNA from all collected cells instead of representative metaphases. The H9 cells were cultured for 12 passages on the JAR culture matrix and Matrigel and analyzed for karyotype changes. At the time of collecting the samples, we also performed flow cytometry analysis on the cells to confirm that the cells were positive for the pluripotency markers SSEA3 and TRA1-60 (data not shown). The H9 cells did not show changes in the karyotype after 12-passage culture on JAR matrix or Matrigel (Figure S2). However, since the karyotyping method does not recognize inversions, translocations or low-level mosaicism, we cannot rule out the possibility of such changes in the cells.

To investigate whether the hPSCs cultured on JAR culture matrix were able to differentiate to endoderm, ectoderm and mesoderm, we performed *in vitro* differentiation by inducing embryoid body formation on ultra low-adherent plates. The hPSCs formed embryoid bodies that contained endodermal (SOX17), mesodermal (VIMENTIN) and ectodermal (BETA(III)TUBULIN/TUJ1) structures (Figure 2d demonstrates the results for HEL11.4. and Figure S3 shows the results for FES29). To find out whether the hPSCs grown on JAR matrix are able to differentiate *in vivo*, we transplanted the hPSCs cultured on JAR matrix into testes of nude mice. Indeed, the transplanted cells formed multiform teratomas containing mature tissues from all the germ layer lineages, as shown for HEL11.4 in Figure 2e. The results for FES29 are shown in Figure S3.

### JAR Matrix Allows hPSC Differentiation into Neuronal and Hepatocyte-like Cells

**Figure 3:**
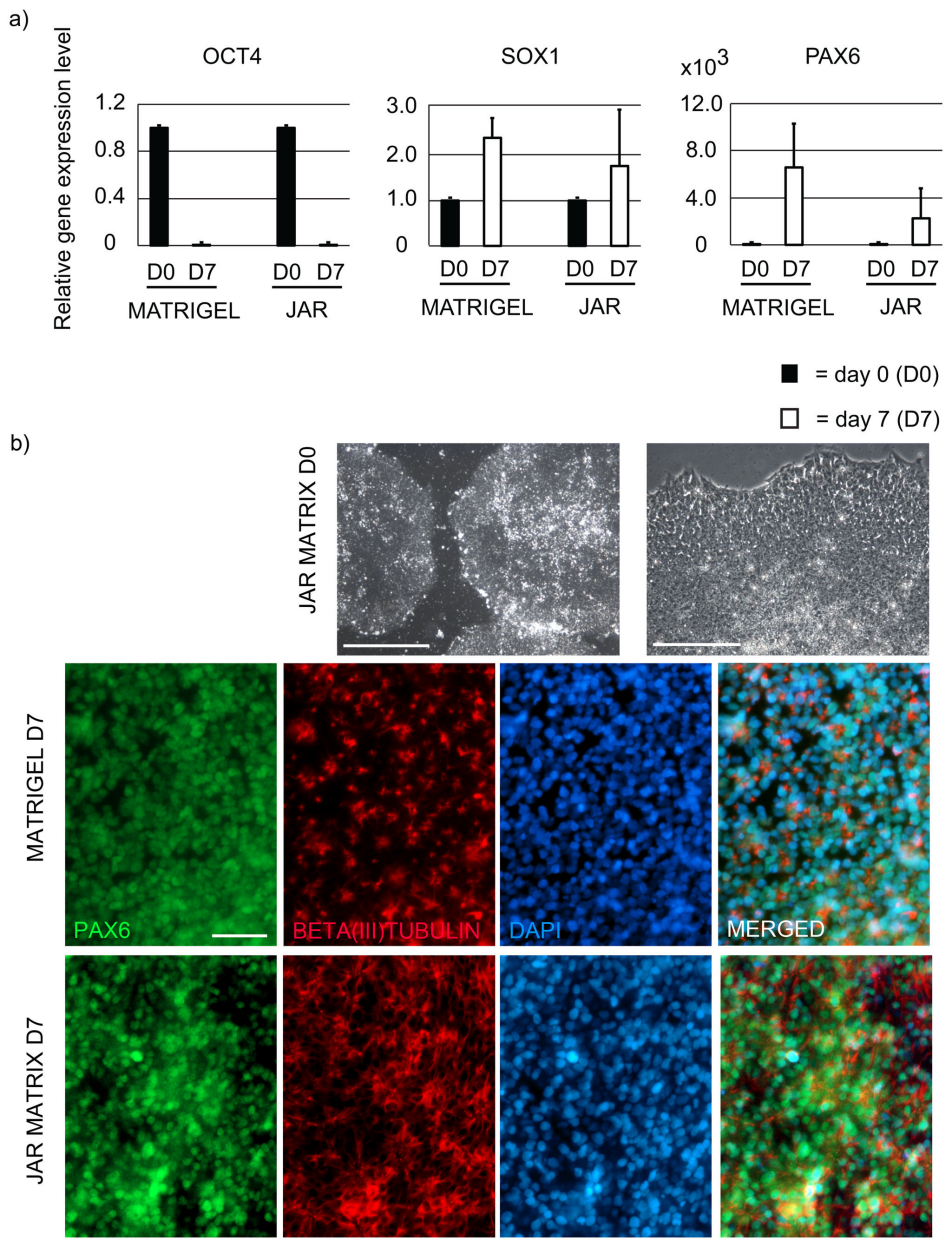
JAR matrix allows hPSC differentiation to neuronal lineages. A) The qPCR results show that the expression level of OCT4 declined during directed neuronal differentiation on JAR matrix and Matrigel while the expression levels of SOX1 and PAX6 increased. Expression level is relative to an in-house prepared positive control mix, which contains cDNA from undifferentiated and differentiated hPSCs and human pancreatic islets. Data represent the mean (±SD) of results from two independent experiments with the hPSC line H9. B) Phase contrast images of the H9 cells cultured on JAR matrix prior to onset of differentiation (upper panel). Scale bars 500 µm (left) and 100 µm, (right). After a 7-day differentiation, the majority of the hPSCs had differentiated to PAX6 and/or BETA(III)TUBULIN positive cells. The data represents the hPSC line H9, cultured for 5 passages on JAR matrix before the differentiation.

We next tested two targeted differentiation protocols with the cells cultured on JAR matrix. We induced differentiation towards neuronal cell fate by using a simple protocol based on two small molecules, Dorsomorphin (also known as Compound C) and SB431542 [24]. During the course of seven-day differentiation, the H9 cells turned into neuronal cells on JAR matrix and on Matrigel, as shown in Figure 3. The qPCR results show pronounced down-regulation of POU5F1, whereas the relative expression level of SOX1 and PAX6 had increased (Figure 3a). The morphology of the H9 cells on JAR matrix prior to onset of differentiation is shown in Figure 3b (the upper panel). After a 7-day differentiation, immunocytochemistry staining for PAX6 and BETA(III)TUBULIN showed abundant neuronal commitment on both culture matrices (Figure 3b; lower panels). These results indicate that most of the cells had differentiated into neuronal lineage (Figure 3).

### JAR Matrix Supports hPSC Differentiation into Hepatocyte-like Cells

**Figure 4:**
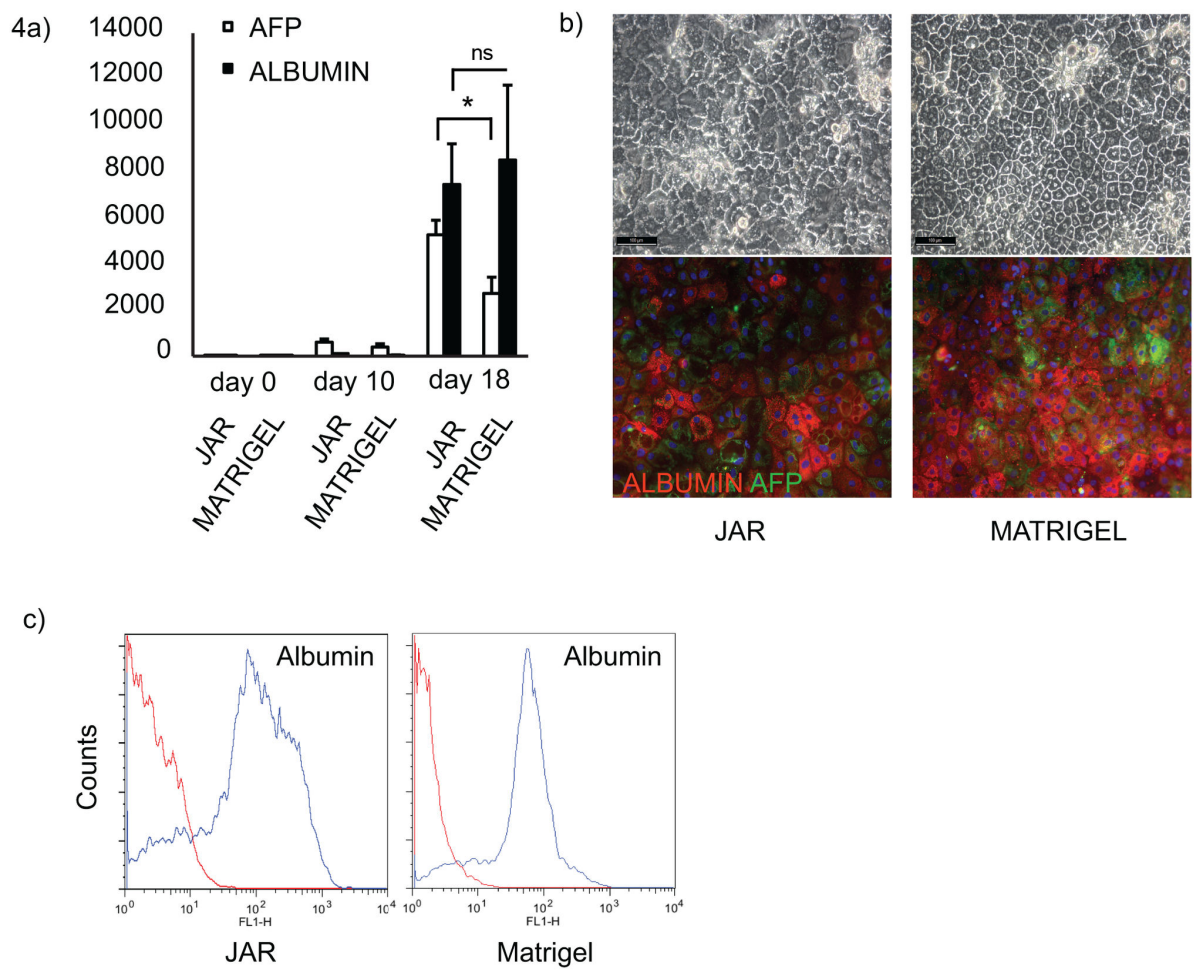
JAR matrix supports hPSC differentiation to hepatocyte like cells. A) qRT-PCR analysis of the expression levels of hepatocyte differentiation markers, AFP and ALBUMIN, during differentiation. Data represent the mean (±SEM) of three independent experiments on H9 and HEL11.4 cells. B) The morphology (upper panel) and immunocytochemistry for ALBUMIN and AFP (lower panel) of the HEL11.4 cells after differentiation. Scale bars 100 µm. C) Quantitative flow cytometry analysis of the cells after differentiation to hepatocyte like cells on JAR matrix or Matrigel. The results are representative for three independent experiments.

The other differentiation protocol, based on the results by Hay and coworkers directs hPSCs to hepatocyte-like cells [20]. The hPSCs were first induced to undergo definitive endoderm differentiation for 5 days, followed by hepatocyte differentiation. There were no significant differences between the definitive endoderm cells differentiated on JAR matrix and on Matrigel (data not shown). After 18 days of differentiation, prominent hepatocyte differentiation had taken place on both culture matrices (Figure 4). ALBUMIN and α-FETOPROTEIN expression levels increased throughout the differentiation, as shown by qPCR (Figure 4a). The α-FETOPROTEIN expression increased 2700-fold on Matrigel and 5200-fold on JAR matrix (p=0.0154) and the relative ALBUMIN expression level of the hepatocyte-like cells differentiated on JAR matrix was 7400-fold, while on Matrigel the relative albumin expression level was 8500-fold (Figure 4a). The difference in albumin expression level was not statistically significant. The typical morphology of the differentiated cells and immunocytochemistry staining for albumin and α-fetoprotein are shown for HEL11.4. in figure 4b. The flow cytometry results confirmed prominent differentiation into hepatocyte-like cell population (Figure 4c). These data show that the JAR culture matrix can be used to differentiate hPSCs into neuroectodermal and endodermal target tissues.

### Effective Derivation of hiPSC Lines on JAR Matrix

**Figure 5:**
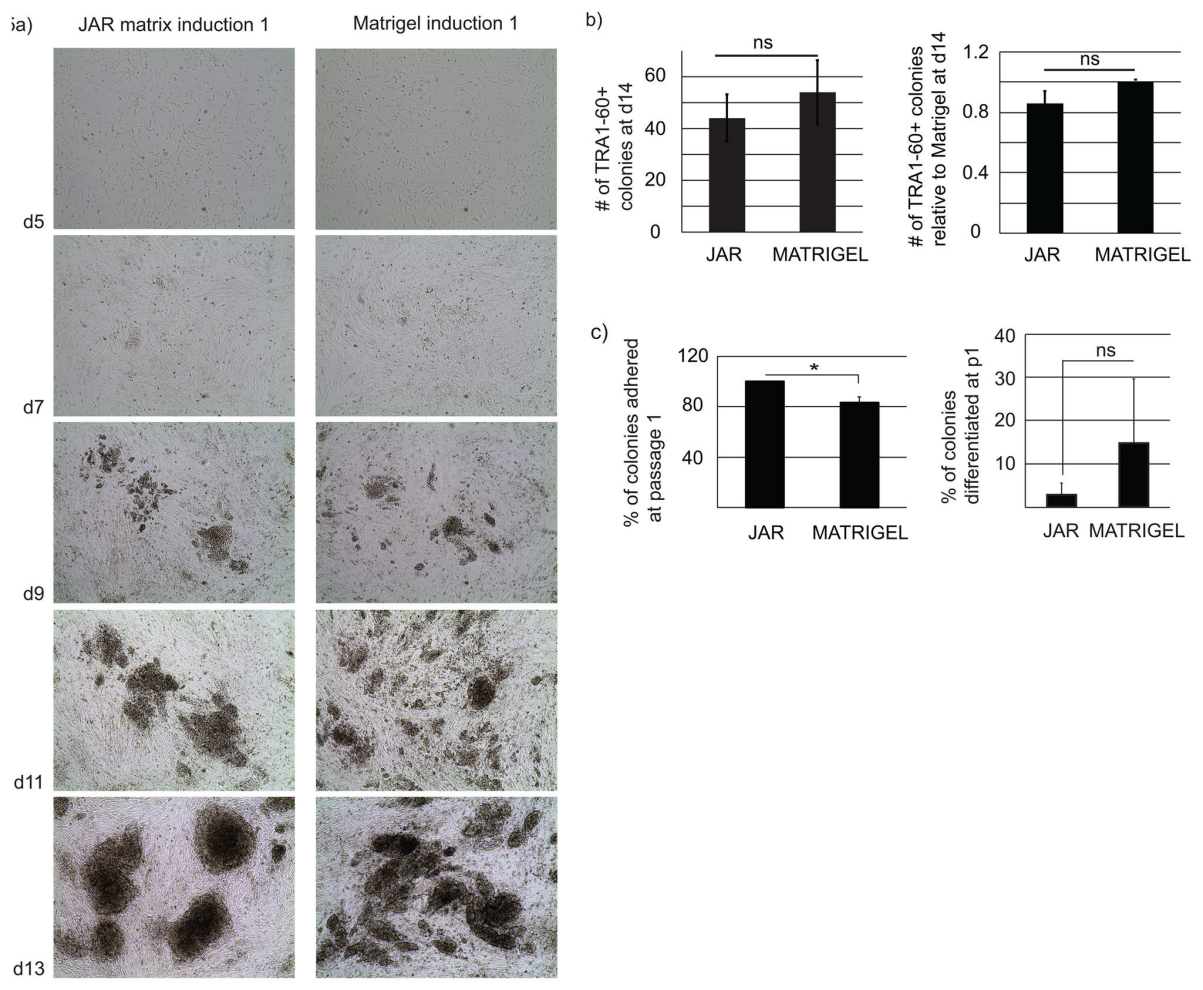
JAR matrix supports adhesion of the early hiPSC colonies. A) Formation of the hiPSC colonies on JAR matrix (left panel) and Matrigel (right panel) at day 5, 7, 9, 11 and 13 after retroviral transduction. Objective magnification 4X. B) The hiPSC colonies from three independent retroviral inductions on JAR matrix and Matrigel were stained for TRA1-60 on day 14 and imaged by the Cell-IQ imaging system. There was no difference in the number of colonies. N=3. C) Twelve clones from three independent inductions were picked and plated either on JAR matrix or Matrigel. The clones adhered significantly better on JAR matrix (left). There was no significant difference on early differentiation of the clones on JAR matrix and Matrigel (right). N=3.

Finally, we tested whether JAR matrix could be used for hiPSC induction and early passaging of the newly formed colonies. Human foreskin fibroblasts (5x10^4^ cells/well) were induced by retroviruses containing OCT4, KLF4, SOX2, and c-myc. Three days after induction the cells were split on either JAR matrix or Matrigel. Morphology of the forming iPS cell colonies was examined daily. There were fewer small ‘satellite’ colonies on JAR matrix than on Matrigel (Figure 5a). The cells were fixed at day 14 post induction and stained for TRA1-60. No difference between the induction efficacy on JAR matrix and Matrigel was observed (Figure 5b and Figure S4). To study the attachment and survival of newly formed hiPSC colonies, we picked twelve colonies from three separate inductions at day 14 and plated them on either JAR matrix or Matrigel. We then counted how many colonies adhered and formed self-renewing clones. All the colonies plated on JAR matrix adhered and formed morphologically typical hiPSC lines, whereas only 83% of the colonies plated on Matrigel adhered (p=0.025; Student’s t-test) (Figure 5c). We evaluated the morphology of the clones on JAR matrix and Matrigel and counted how many of the adhered clones maintained undifferentiated morphology. There was no statistically significant difference between the matrices in the sustainability of the early hiPSC clones.

**Figure 6:**
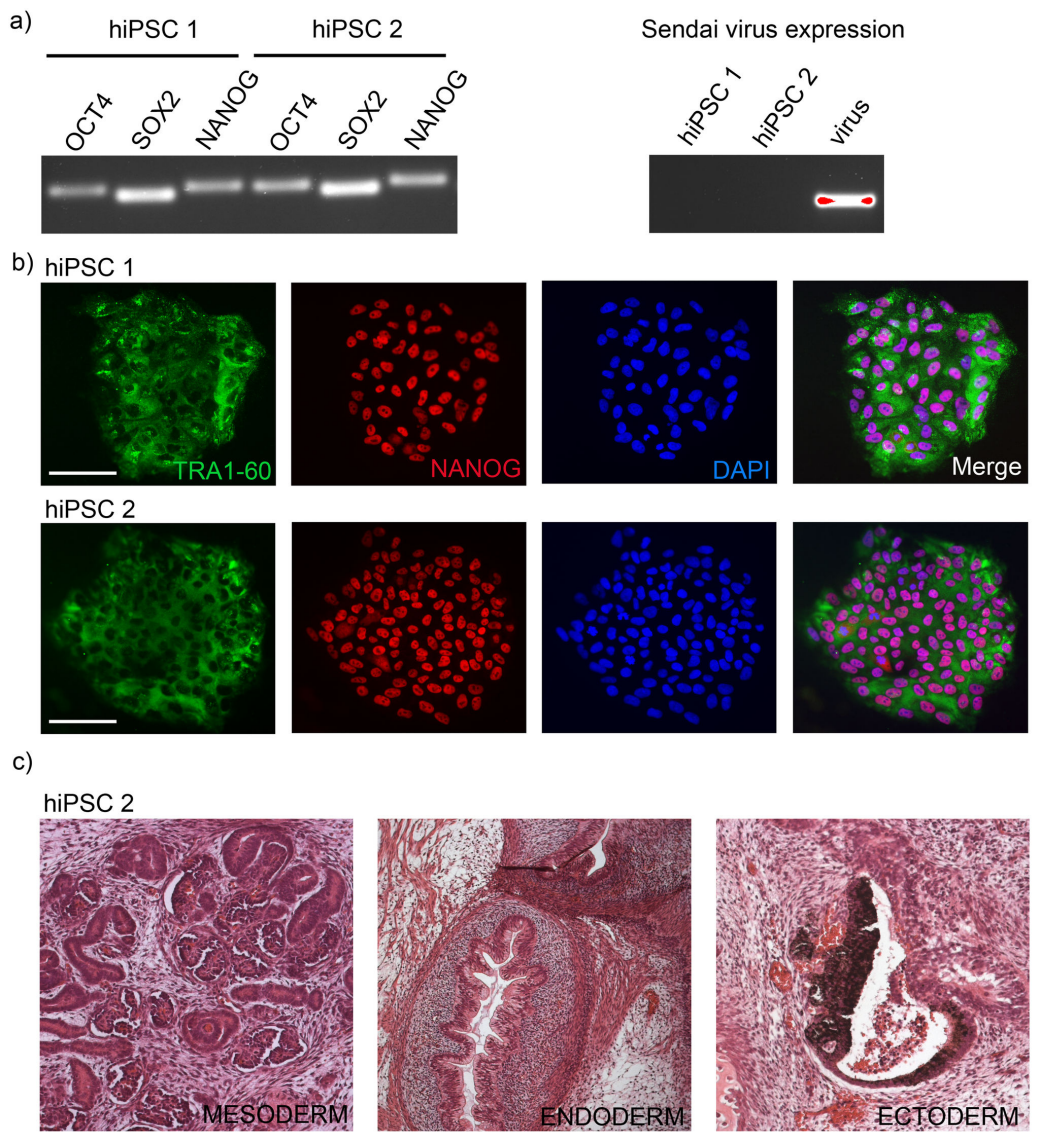
Characterization of new hiPSC lines generated on JAR-matrix. A) Human foreskin fibroblasts were induced to pluripotency by the Sendai virus reprogramming kit. Both hiPSC lines showed endogenous expression of OCT4, SOX2 and NANOG but were free from sendai virus replicon when analyzed at passage 12. B) The hiPSC lines generated on JAR matrix were stained for presence of TRA1-60 and NANOG. Both of the hiPSC lines were positive for the pluripotency markers. Scale bar 100 µm. C) Undifferentiated hiPSC2 cells were transplanted into testes of nude mouse. The hiPSC 2 cells had formed well-matured teratoma, which contained endodermal, mesodermal and neuroectodermal derivatives.

Next, we established new hiPSC lines on JAR matrix by using non-integrating Sendai viruses. Two to three weeks after transductions individual newly formed hiPSC colonies were picked on JAR matrix (passage 1). Two hiPSC lines were cultured on JAR matrix for 15 passages and characterized between passages 12 and 15. The new hiPSC lines met all of the standard criteria for pluripotent cells. They expressed high levels of POU5F1, SOX2 and NANOG but were free from transgene expression, as measured by RT-PCR (Figure 6a). They were positive for TRA1-60 and NANOG (Figure 6b). Undifferentiated cells of the hiPSC2 line formed well-differentiated teratomas containing tissue from mesodermal, endodermal and ectodermal lineages when transplanted into nude mice (Figure 6c). Their karyotypes were normal by the KaryoLite method (Figure S5).

## Discussion

There has been a growing interest in developing defined, serum-free culture conditions for hPSCs, based on distinct basement membrane protein coatings and defined culture media [12-15,25-27]. However, a major disadvantage in the use of the purified and/or recombinant basement membrane proteins is their limited availability and high cost. Therefore, by utilizing a commercially available human cell line that, among other ECM components, produces high quantities of laminins 511 and -111, we have established an economical and easy-to-prepare culture matrix, which supports the growth of undifferentiated hPSCs and development of early hiPSC clones. We were able to maintain all the tested hPSC lines on the JAR culture matrix for at least 15 passages using a defined, serum-free cell culture medium. The hPSC lines maintained high expression levels of the typical hPSC marker genes and they were able to differentiate into all germ line derivatives when induced. These data show that the JAR culture system allows the maintenance and expansion of the undifferentiated cells without restricting their differentiation capacity. The hPSCs cultured on JAR matrix formed very well differentiated, mature teratomas and differentiated spontaneously in embryoid body assay. In addition, we were able to differentiate the hPSCs cultured on JAR matrix to neural as well as to hepatocyte-like cells, demonstrating that the matrix can be utilized in targeted differentiation of hPSCs.

Novel hiPSC induction methods are developed continuously. However, a standard practice is still to pick the newly formed colonies on a feeder cell layer. We were able to generate new hiPSC lines on JAR culture matrix. Importantly, while there was no difference in hiPSC induction efficacy, the newly generated hiPSCs adhered better on JAR matrix than on Matrigel. Enhanced adhesion of the early hiPSCs may serve to increase the quality of the established clones by reducing partially reprogrammed and aberrant clones.

Fukusumi et al. recently reported derivation of hiPSCs on pericellular matrix of decidua derived mesenchymal cells [28]. They showed that the decidua derived mesenchymal cell matrix supported derivation and long-term culture of hiPSCs. However, they did not characterize the extracellular matrix produced by the mesenchymal cells. In addition, the hPSCs showed some differentiation after long-term culture on decidua derived mesenchymal cell matrix when cultured in defined culture medium, StemPro, but not when cultured in mEF-conditioned medium. Our recent data suggests that mEFs produce lm-511 and it is known that lm-511 synthesis correlates with the ability of human fibroblasts to support hPSC maintenance [29]. Therefore, we suggest that the presence of lm-511 in JAR matrix supports the self-renewal of hPSCs.

hPSCs are prone to apoptosis when dissociated for example at passaging and therefore, Rho associated protein kinase (ROCK) inhibitors are widely used [30]. Based on our results, ROCK inhibitors are not needed to maintain hPSC viability on JAR matrix. Similar results were recently reported for the laminin E8 fragment, comprising the integrin-binding C-terminal domains of either lm-511 or -332 [31]. Hence, we suggest that JAR matrix supports the adhesion and self-renewal of human pluripotent stem cells by providing an ideal ECM milieu, rich in physiologically relevant laminin isoforms. In conclusion, our results indicate that in addition to supporting the self-renewal of pre-existing hPSC lines, JAR culture matrix is ideal for the support of newly derived hiPSC colonies.

## Supporting Information

Figure S1 **Representative results from the flow cytometry analysis.** The hPSC lines HEL11.4 and FES29 were analyzed for presence of stem cell markers TRA1-60, SSEA3 and H type 1 antigen and differentiation marker SSEA1, after 15-passage culture on JAR matrix. White filling indicates the negative control and black filling indicates staining with the primary antibody, followed with the secondary antibody.
(TIF)

Figure S2 **hESC line H9 retained a normal karyotype after cultured on either JAR matrix or Matrigel for 12 passages.** In the figure is the representative data from the karyotypic analysis of the H9 cells grown on either JAR matrix or Matrigel. The red and blue lines indicate the normalized chromosomal signal ratios against the female (red) and male (blue) references with normal genotype as calculated by BoBs software. For the normal chromosomes the signal ratios should reside inside the reference area around value 1, whereas in the case of chromosomal abbreviation both signal ratios should exceed the calculated threshold values and locate clearly outside the calculated reference area.
(TIF)

Figure S3 **JAR matrix supports pluripotency of hPSCs.** A) The phase contrast image of the hPSC line FES29 cultured on JAR matrix for 15 passages.
B) Undifferentiated cells of the hPSC line FES29 were transplanted into nude mouse for teratoma formation after 15-passage culture on JAR matrix. The cells formed derivatives of mesoderm (VIMENTIN), ectoderm (BETA(III)TUBULIN/TUJ1) and endoderm (SOX17), indicating that the cells had maintained pluripotent.
C) The hPSC line FES29 formed embryoid bodies with derivatives from all germ layers: endoderm (FOXA2), mesoderm (BRACHYURY), and ectoderm (BETA(III)TUBULIN/TUJ1) after 15-passage culture on JAR matrix. Scale bars 100µm.
(TIF)

Figure S4 **The JAR matrix supports human iPSC inductions.** Three independent, retroviral hiPSC inductions were performed on JAR matrix and Matrigel. Induction efficiencies were determined by counting the alkaline phosphatase positive colonies at day 14. The iPSC induction efficiency on JAR matrix was comparable to that on Matrigel. Data represent the mean (±SEM) of three independent inductions.
(TIF)

Figure S5 **Two new hiPSC lines generated and cultured on JAR matrix maintained normal karyotypes.** hiPSC1 and hiPSC2 lines were generated on the JAR matrix. Both of the new hiPSC lines showed normal karyotype after cultured for 12 passages on JAR matrix. The red and blue lines indicate the normalized chromosomal signal ratios against the female (red) and male (blue) references with normal genotype as calculated by BoBs software. For the normal chromosomes the signal ratios should reside inside the reference area around value 1, whereas in the case of chromosomal abbreviation both signal ratios should exceed the calculated threshold values and locate clearly outside the calculated reference area.
(TIF)

methods S1 **Supplementary Materials and Methods.**
(PDF)
