# Supplementary figures and images for: A Novel Feeder-Free Culture System for Human Pluripotent Stem Cell Culture and Induced Pluripotent Stem Cell Derivation
Source: PLoS One. 2013 Oct 2;8(10):e76205. doi: 10.1371/journal.pone.0076205 (PMC3788803; doi:10.1371/journal.pone.0076205)

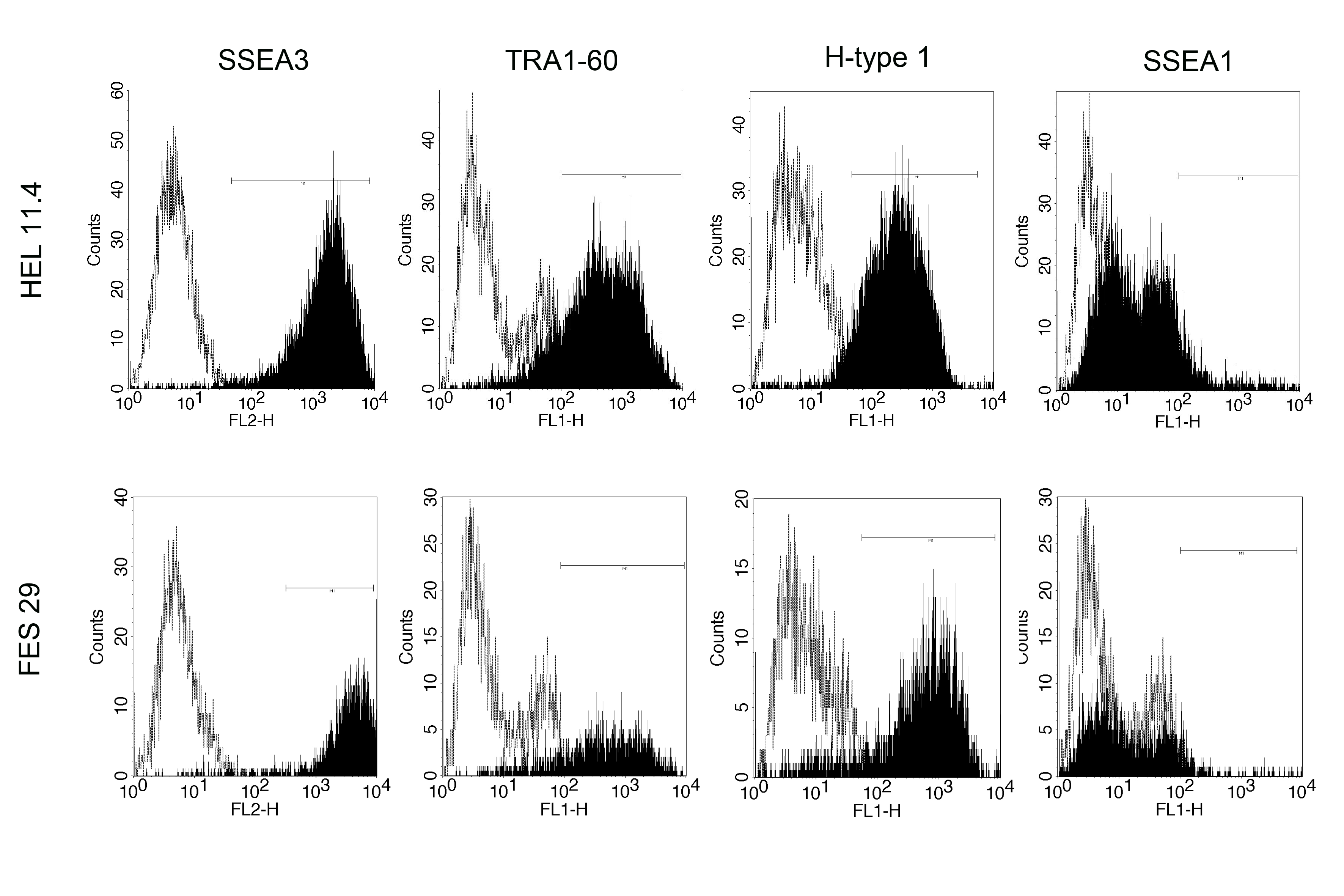

Supplement: Figure S1 — Representative results from the flow cytometry analysis. The hPSC lines HEL11.4 and FES29 were analyzed for presence of stem cell markers TRA1-60, SSEA3 and H type 1 antigen and differentiation marker SSEA1, after 15-passage culture on JAR matrix. White filling indicates the negative control and black filling indicates staining with the primary antibody, followed with the secondary antibody. (TIF) [file pone.0076205.s001.tif]

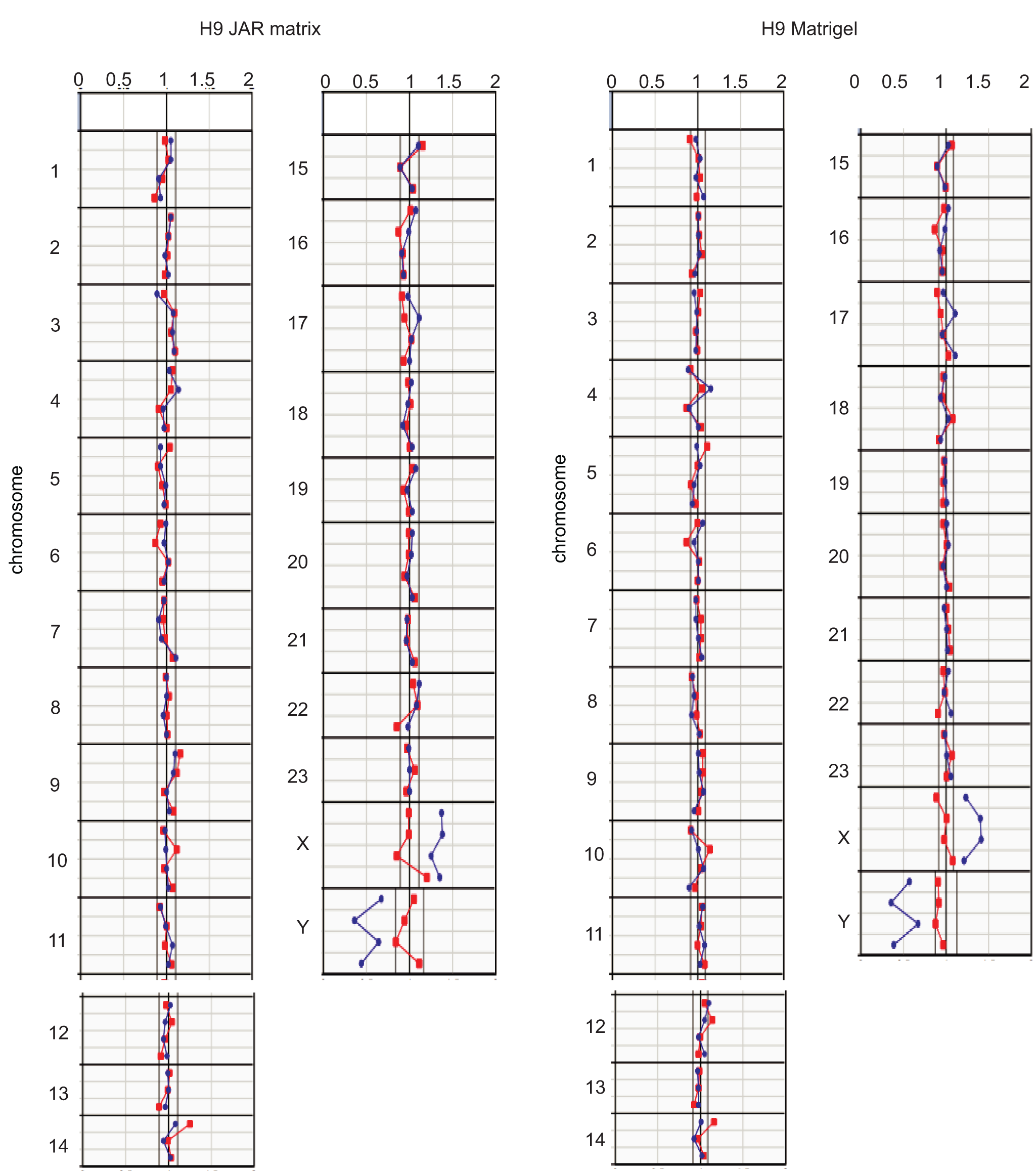

Supplement: Figure S2 — hESC line H9 retained a normal karyotype after cultured on either JAR matrix or Matrigel for 12 passages. In the figure is the representative data from the karyotypic analysis of the H9 cells grown on either JAR matrix or Matrigel. The red and blue lines indicate the normalized chromosomal signal ratios against the female (red) and male (blue) references with normal genotype as calculated by BoBs software. For the normal chromosomes the signal ratios should reside inside the reference area around value 1, whereas in the case of chromosomal abbreviation both signal ratios should exceed the calculated threshold values and locate clearly outside the calculated reference area. (TIF) [file pone.0076205.s002.tif]

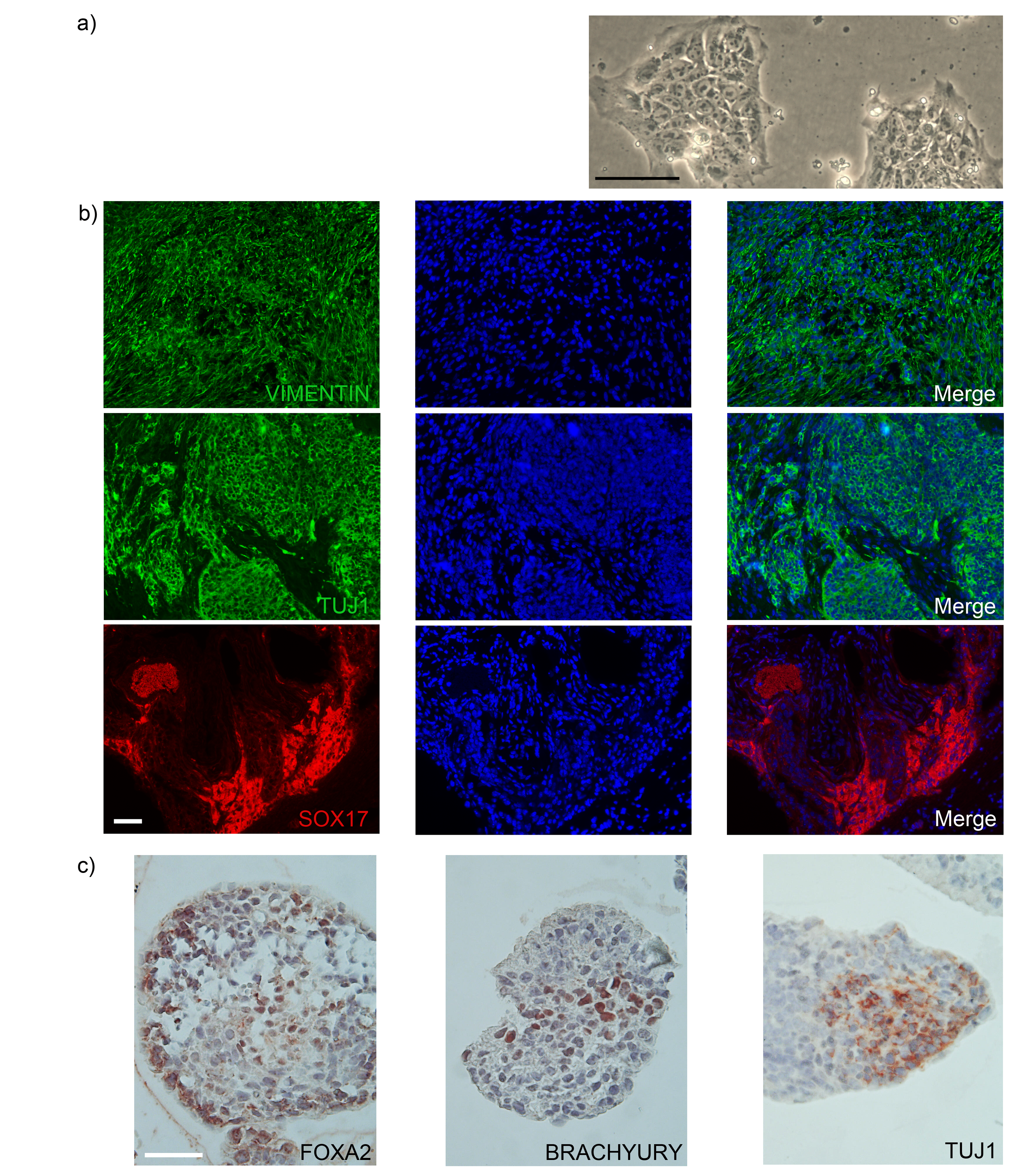

Supplement: Figure S3 — JAR matrix supports pluripotency of hPSCs. A) The phase contrast image of the hPSC line FES29 cultured on JAR matrix for 15 passages. B) Undifferentiated cells of the hPSC line FES29 were transplanted into nude mouse for teratoma formation after 15-passage culture on JAR matrix. The cells formed derivatives of mesoderm (VIMENTIN), ectoderm (BETA(III)TUBULIN/TUJ1) and endoderm (SOX17), indicating that the cells had maintained pluripotent. C) The hPSC line FES29 formed embryoid bodies with derivatives from all germ layers: endoderm (FOXA2), mesoderm (BRACHYURY), and ectoderm (BETA(III)TUBULIN/TUJ1) after 15-passage culture on JAR matrix. Scale bars 100µm. (TIF) [file pone.0076205.s003.tif]

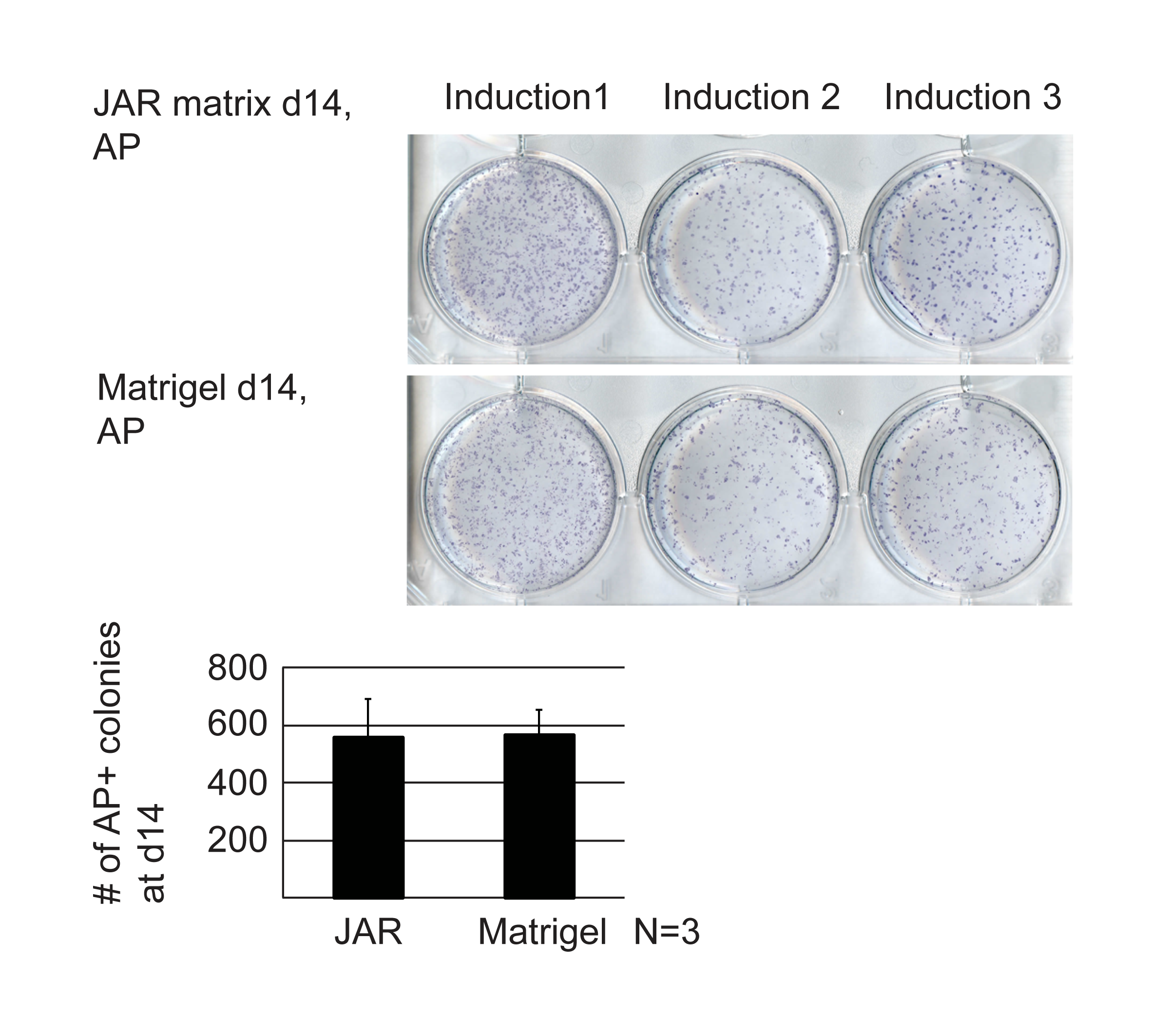

Supplement: Figure S4 — The JAR matrix supports human iPSC inductions. Three independent, retroviral hiPSC inductions were performed on JAR matrix and Matrigel. Induction efficiencies were determined by counting the alkaline phosphatase positive colonies at day 14. The iPSC induction efficiency on JAR matrix was comparable to that on Matrigel. Data represent the mean (±SEM) of three independent inductions. (TIF) [file pone.0076205.s004.tif]

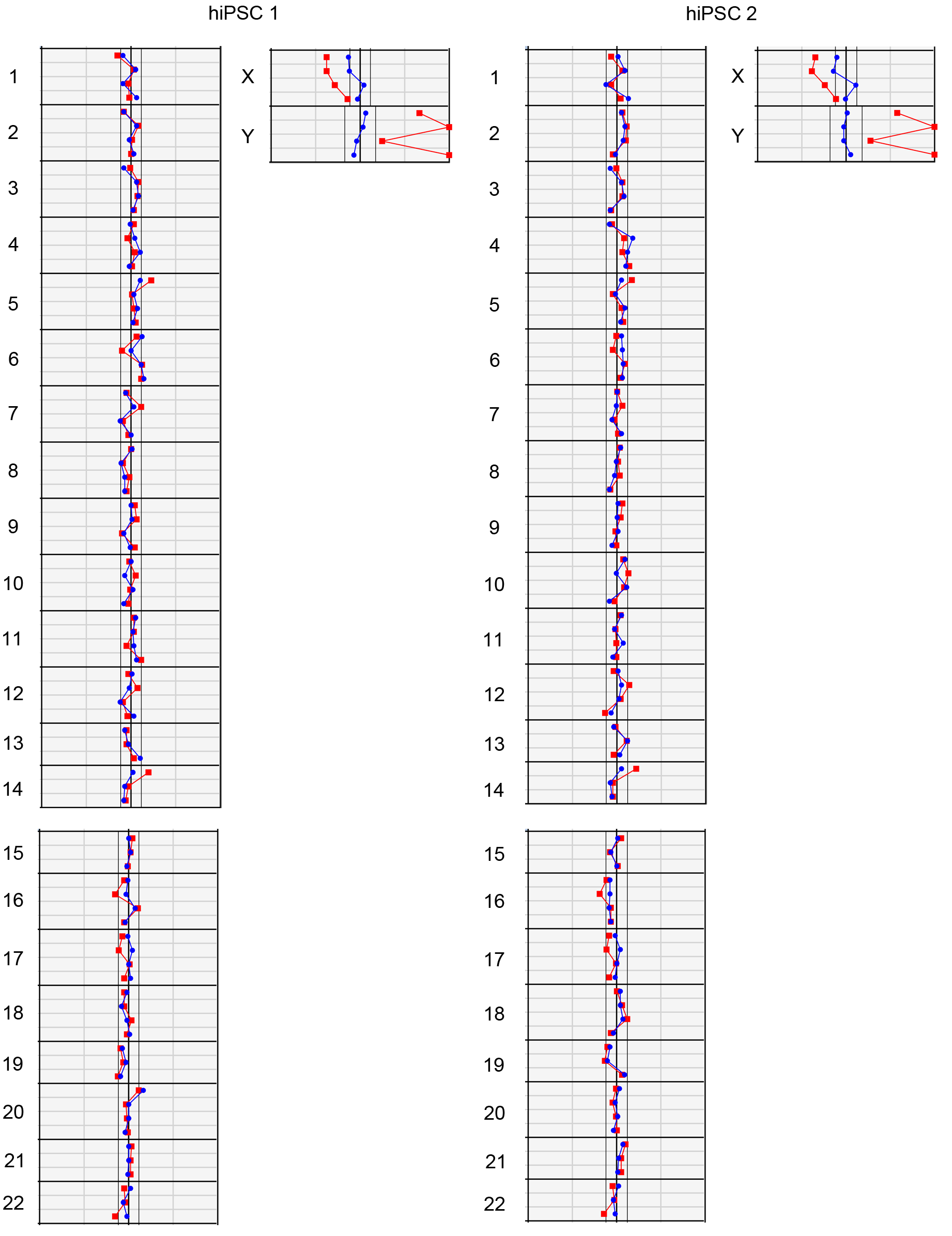

Supplement: Figure S5 — Two new hiPSC lines generated and cultured on JAR matrix maintained normal karyotypes. hiPSC1 and hiPSC2 lines were generated on the JAR matrix. Both of the new hiPSC lines showed normal karyotype after cultured for 12 passages on JAR matrix. The red and blue lines indicate the normalized chromosomal signal ratios against the female (red) and male (blue) references with normal genotype as calculated by BoBs software. For the normal chromosomes the signal ratios should reside inside the reference area around value 1, whereas in the case of chromosomal abbreviation both signal ratios should exceed the calculated threshold values and locate clearly outside the calculated reference area. (TIF) [file pone.0076205.s005.tif]
