# Supplementary material for: A Novel Feeder-Free Culture System for Human Pluripotent Stem Cell Culture and Induced Pluripotent Stem Cell Derivation
Source: PLoS One. 2013 Oct 2;8(10):e76205. doi: 10.1371/journal.pone.0076205 (PMC3788803; doi:10.1371/journal.pone.0076205)
Supplement: methods S1 — Supplementary Materials and Methods. (PDF) [file pone.0076205.s006.pdf]

Supplementary Materials and Methods

KARYOTYPE ANALYSIS

Karyotypes were analyzed by KaryoLite method in The Finnish Microarray and Sequencing Center, Turku Centre for Biotechnology, Helsinki, Finland. The assay measures DNA copy numbers at the chromosome arm resolution utilizing bacterial artificial chromosome (BAC) probes immobilized onto color-encoded polystyrene microspheres distinguishable by fluorometry. Genomic DNA was isolated by The GenElute Mammalian Genomic DNA Purification Kit and 500ng of genomic DNA from each sample was sent for KaryoLite-analysis.

The red and blue lines in the figures indicate the normalized chromosomal signal ratios against the female (red) and male (blue) references with normal genotype as calculated by BoBs™ software. For the normal chromosomes the signal ratios should reside inside the reference area around value 1, whereas in the case of chromosomal abbreviation both signal ratios should exceed the calculated threshold values and locate clearly outside the calculated reference area.

IMMUNOHISTOCHEMISTRY

FES29 embryoid bodies were washed twice with 1X PBS, fixed with 4% PFA for 20 minutes at room temperature and washed several times with 1X PBS. Then, the EBs were embedded into 2% agarose gels, dehydrated and embedded into paraffin. The sections were immunostained for goat FOXA2 (1:1000 in 0.1% Tween-PBS, M20;SantaCruz Biotechnology), rabbit BRACHYURY (1:1000 in 0.1% Tween-PBS, H-210;SantaCruz Biotechnology) and mouse BETA(III)TUBULIN/TUJ1 (1:5000 in 0.1% Tween-PBS, MAB1195; R&D Systems) for over night at 4°C, washed and treated with secondary antibodies (biotinylated anti-goat, biotinylated anti-rabbit and biotinylated anti-mouse, respectively, all 1:200 in 0.1% Tween-PBS, for 30 minutes at RT in dark. Then, the sections were washed, treated with streptavidin horseradish conjugate (1:200 in 0.1% Tween-PBS) for 30 minutes at RT, in dark, washed again and treated with AEC substrate from eight to twenty five minutes, in dark after which the reactions were stopped by deionized water.

ALKALINE PHOSPHATASE ASSAY

The cells were washed and fixed with 4% PFA for 10 minutes at room temperature. Then, the cells were washed three times with 1X PBS and treated with alkaline phosphatase detection solution (2% NBT/BCIP (Roche Applied Science) in 100mM Tris-HCl, pH9.5; 100mM NaCl, 50mM $MgCl_2$).
